# Bcl-2 Expression in Pericytes and Astrocytes Impacts Vascular Development and Homeostasis

**DOI:** 10.1038/s41598-019-45915-4

**Published:** 2019-07-04

**Authors:** Ismail S. Zaitoun, Catherine M. Wintheiser, Nasim Jamali, Shoujian Wang, Andrew Suscha, Soesiawati R. Darjatmoko, Katherine Schleck, Barbara A. Hanna, Volkhard Lindner, Nader Sheibani, Christine M. Sorenson

**Affiliations:** 1 Department of Ophthalmology and Visual Sciences, University of Wisconsin School of Medicine and Public Health, Madison, WI USA; 2 McPherson Eye Research Institute, University of Wisconsin School of Medicine and Public Health, Madison, WI USA; 3 Department of Pediatrics, University of Wisconsin School of Medicine and Public Health, Madison, WI USA; 4 Center for Molecular Medicine, Maine Medical Center Research Institute, Scarborough, ME USA

**Keywords:** Apoptosis, Cell lineage

## Abstract

B-cell lymphoma 2 (Bcl-2) protein is the founding member of a group of proteins known to modulate apoptosis. Its discovery set the stage for identification of family members with either pro- or anti-apoptotic properties. Expression of Bcl-2 plays an important role during angiogenesis by influencing not only vascular cell survival, but also migration and adhesion. Although apoptosis and migration are postulated to have roles during vascular remodeling and regression, the contribution of Bcl-2 continues to emerge. We previously noted that the impaired retinal vascularization and an inability to undergo pathologic neovascularization observed in mice globally lacking Bcl-2 did not occur when mice lacked the expression of Bcl-2 only in endothelial cells. To further examine the effect of Bcl-2 expression during vascularization of the retina, we assessed its contribution in pericytes or astrocytes by generating mice with a conditional Bcl-2 allele (Bcl-2^Flox/Flox^) and Pdgfrb-cre (Bcl-2^PC^ mice) or Gfap-cre (Bcl-2^AC^ mice). Bcl-2^PC^ and Bcl-2^AC^ mice demonstrated increased retinal vascular cell apoptosis, reduced numbers of pericytes and endothelial cells and fewer arteries and veins in the retina. Bcl-2^PC^ mice also demonstrated delayed advancement of the superficial retinal vascular layer and aberrant vascularization of the deep vascular plexus and central retina. Although pathologic neovascularization in oxygen-induced ischemic retinopathy (OIR) was not affected by lack of expression of Bcl-2 in either pericytes or astrocytes, laser-induced choroidal neovascularization (CNV) was significantly reduced in Bcl-2^PC^ mice compared to littermate controls. Together these studies begin to reveal how cell autonomous modulation of apoptosis in vascular cells impacts development and homeostasis.

## Introduction

The term apoptosis describes a process by which cells determine their demise. Over 45 years ago Kerr and colleagues adopted this term, identifying apoptotic cells during normal embryonic development and in malignant neoplasms^1,2^. Apoptosis has a central role during organ development and maintenance by facilitating clearance of cells that are no longer needed or dysfunctional. Its aberrant regulation can be attributed to various developmental abnormalities including cleft palate, syndactyly^3^ and renal hypoplasia^4^.

Apoptosis can be initiated by both intrinsic and extrinsic stimuli. The intrinsic or mitochondrial pathway relies on a balance of anti-apoptotic proteins such as Bcl-2 and pro-apoptotic family members. Bcl-2 is a novel proto-oncogene and is the founding factor of a group of proteins that impact apoptosis levels both during development and in disease states. Bcl-2 is inserted into the outer mitochondrial, endoplasmic reticulum and nuclear membranes with the bulk of the protein facing the cytosol, potentially available to interact with other proteins^5^. Members of the anti-apoptotic family of proteins in mammals have up to four Bcl-2 homology (BH) common domains and a transmembrane spanning region at the carboxyl end of the protein^6^. Overexpression of Bcl-2 blocks apoptosis normally triggered by a diverse group of agents while cells lacking Bcl-2 are more susceptible to apoptosis by these agents. Thus, modulating Bcl-2 expression could impact apoptosis levels influencing development or disease progression.

Angiogenesis plays a central role during development and its aberrant regulation contributes to various diseases including ocular disease with a neovascular component. Multiple vascular cell types play an integral role in orchestrating the development of the retinal vasculature. In the mouse, formation of the retinal vasculature is initiated after birth following a path laid out by astrocytes to form the superficial layer. These vessels sprout deep into the inner retina and form the deep and intermediate retinal vascular plexus. Pericytes cover these vessels thus protecting endothelial cells and facilitating maintenance of competent microvessel function. More importantly, pericytes not only play roles during development but also are essential for stabilization, maturation and remodeling of the vasculature^7^. Thus, the interchange among astrocytes, pericytes and endothelial cells is essential for retinal vascular development and sustaining a mature vascular phenotype^8–10^.

The impact modulating survival of specific retinal vascular cells has during eye development and disease is not well understood. Our prior studies showed that global loss of Bcl-2 impaired vascularization of the retina as evidenced by decreased retinal endothelial cell, pericyte and retinal artery numbers, delayed advancement of the superficial layer and decreased vascularity of the superficial and deep vascular plexus as well as attenuation of pathologic neovascularization during oxygen-induced ischemic retinopathy (OIR) in mice^11^. Since Bcl-2 plays an essential role during angiogenesis, we previously examined the role endothelial cell Bcl-2 expression plays during retinal vascularization. We found that expression absence of Bcl-2 in endothelial cells did not have as profound an impact on retinal vascular development or pathologic neovascularization associated with OIR as we observed previously in global Bcl-2 −/− mice^11,12^. Thus, Bcl-2 expression in retinal vascular cell type(s) other than endothelial cells may be critical for appropriate retinal vascularization.

Here we addressed the cell autonomous impact Bcl-2 expression has in developmental and pathological retinal and choroidal neovascularization by generating mice carrying a conditional Bcl-2 allele (Bcl-2^Flox/Flox^) and Pdgfrb-cre (Bcl-2^PC^ mice) or Gfap-cre (Bcl-2^AC^ mice). Spreading of the retinal superficial layer was delayed in Bcl-2^PC^ compared to Bcl-2^Flox/Flox^ littermates though it was similar in Bcl-2^Flox/Flox^ and Bcl-2^AC^ mice. Mice lacking Bcl-2 expression in pericytes or astrocytes had decreased artery and vein numbers. Lack of central vessels in the optic papilla were noted in retinas from Bcl-2^PC^ but not Bcl-2^Flox/Flox^ or Bcl-2^AC^ mice. Bcl-2^PC^ and Bcl-2^AC^ mice showed reduced numbers of both endothelial cells and pericytes. Increased apoptosis and proliferation of endothelial cells, pericytes and astrocytes was noted in the retinal vasculature of Bcl-2^PC^ mice compared with control littermates. A modest increase in endothelial cell and astrocyte apoptosis was noted in retinas from Bcl-2^AC^ mice. We also assessed whether pathologic retinal neovascularization in OIR and laser-induced choroidal neovascularization (CNV) relied on expression of Bcl-2 in pericytes or astrocytes. Although Bcl-2^PC^ mice demonstrated reduced CNV levels compared with littermate controls, we did not observe protection from ischemia-driven neovascularization in retina in either Bcl-2^PC^ or Bcl-2^AC^ mice as we had reported in global Bcl-2 −/− mice^11^. Together these studies further aid our understanding as to how Bcl-2 expression in vascular cells impacts development and pathologic ocular neovascularization. This knowledge will allow us to better exploit its modulation for therapeutic benefit in the eye.

## Results

### Reduced numbers of retinal endothelial cells and pericytes in Bcl-2^PC^ and Bcl-2^AC^ mice

**Figure 1:**
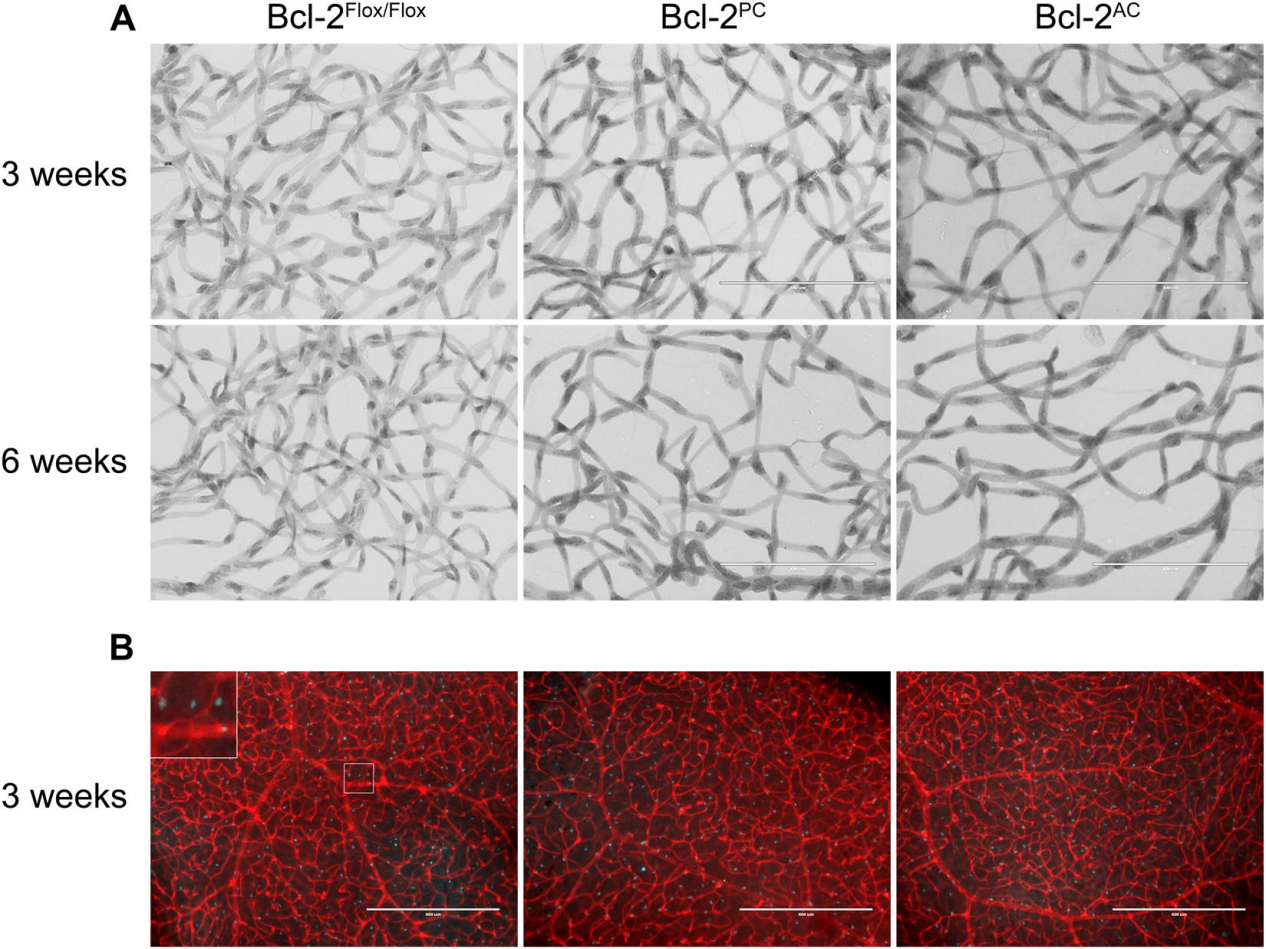
Decreased numbers of retinal endothelial cells and pericytes in conditional Bcl-2 mice. In Panel A, retinas from 3 week and 6 week old mice were prepared by trypsin digest followed by HE/PAS staining and quantification of endothelial cells and pericytes per x400 field of view. Scale bar = 100 µm. In Panel B, retinas from 3 week old mice were stained with anti-Pax2 (blue) and Isolectin B4 (red) and astrocytes quantified. The inset magnifies the boxed area of interest 300%. Scale bar = 400 µm. Four quadrants per eye were counted from > 6 eyes at each time point for the quantitative assessment of this data which is summarized in Table 1.

During development of the retinal vasculature an astrocytic network is initially laid down for the endothelial cells and pericytes to follow forming the nascent retinal vasculature. We had previously shown that in addition to its traditional role inhibiting apoptosis, Bcl-2 expression also influences cell migration, adhesion and extracellular matrix production^13–15^. Therefore, it is tempting to speculate that Bcl-2 expression in astrocytes impacts retinal vascularization. Here we investigated whether expression of Bcl-2 in astrocytes influenced retinal pericyte and endothelial cell numbers at 3 weeks of age or later following retinal vascular remodeling (6 weeks of age). Endothelial cell nuclei are large, oval, and weakly stained and protrude luminally are located within the vessel wall. While pericyte nuclei, which protrude laterally from the vessel wall, are darkly stained, small, and round. At 3 weeks of age in Bcl-2^AC^ mice, pericyte and endothelial cell numbers were significantly lower than their Bcl-2^Flox/Flox^ counterparts (Fig. 1A and Table 1). The reduced numbers of pericytes and endothelial cells were maintained at 6 weeks of age in Bcl-2^AC^ mice (Fig. 1A and Table 1). Thus, lack of expression of Bcl-2 in astrocytes influences both pericyte and endothelial cell numbers in the retinal vasculature.

**Table 1:** Retinal Vascular Cell Numbers.

	Age	Bcl-2^Flox/Flox^	Bcl-2^PC^	Bcl-2^AC^
Pericytes (PC)	P21	37.29 ± 0.96	30.83 ± 0.94^****^	23.58 ± 1.16^*****^
Endothelial Cell (EC)	P21	157.32 ± 3.11	107.92 ± 3.35^*****^	96.54 ± 2.48^*****^
Pericytes (PC)	P42	30.03 ± 0.64	24.1 ± 0.93^*****^	22.8 ± 0.75^*****^
Endothelial Cell (EC)	P42	119.44 ± 2.79	100.00 ± 2.80^****^	91.3 ± 1.94^*****^
**Number of Cells Per High Power Field at (x400)**				
Astrocytes (AC)	P21	224.0 ± 2.1	222.9 ± 2.9	219.0 ± 2.1
**Number of Astrocytes Per Field at (x100)**				

The P values were calculated by comparing samples from Bcl-2^Flox/Flox^ to Bcl-2^PC^ or Bcl-2^AC^ mice at the ages noted: *P < 0.05; **P < 0.01; ***P < 0.001; ****P < 0.0001; and *****P < 0.00001.

Since pericytes stabilize and protect the vasculature, we next addressed whether Bcl-2 expression in pericytes impacts retinal pericyte or endothelial cell numbers. At 3 and 6 weeks of age, pericyte and endothelial cell numbers were reduced in Bcl-2^PC^ mice compared to Bcl-2^Flox/Flox^ mice (Figure 1A and Table 1). Following remodeling at 6 weeks of age, a further decline in retinal endothelial cell and pericyte numbers was noted in Bcl-2^Flox/Flox^ and Bcl-2^PC^ mice. To address whether astrocyte numbers were impacted in mice lacking Bcl-2 expression in pericytes or astrocytes, retinas from P21 mice were wholemount stained with anti-Pax2 and the numbers of Pax2 positive cells counted and noted as astrocytes (Fig. 1B and Table 1). Similar numbers of astrocytes were observed in retinas from Bcl-2^Flox/Flox^, Bcl-2^PC^ and Bcl-2^AC^ mice. These data further demonstrate the impact modulating retinal vascular cell autonomous survival and/or function has on the development and integrity of the retinal vasculature.

### Lack of Bcl-2 in pericytes and astrocytes decreased retinal artery and vein number

**Figure 2:**
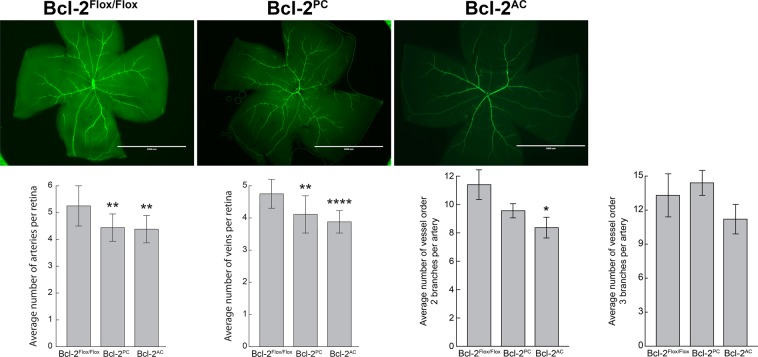
Decreased numbers of retinal arteries and veins in Bcl-2^PC^ and Bcl-2^AC^ mice. Retinas from P21 Bcl-2^Flox/Flox^, Bcl-2^PC^ and Bcl-2^AC^ mice were wholemount stained with anti-α-smooth muscle actin to identify major arteries. The number of arteries and veins per retina were quantified. The average number of vessel order 2 branches and vessel order 3 branches per artery was determined (*P < 0.05, **P < 0.01, ***P < 0.001, ****P < 0.0001; n = 8). Scale bar = 2000 µm. Please note mice conditionally lacking Bcl-2 in pericytes or astrocytes have decreased numbers or retinal arteries and veins.

Here we asked whether Bcl-2 expression in pericytes or astrocytes influenced artery and vein numbers. In Fig. 2, retinas from postnatal day 21 (P21) Bcl-2^Flox/Flox^, Bcl-2^PC^ and Bcl-2^AC^ mice were whole-mount stained for smooth muscle actin (SMA). This stains the major arteries in the retina. Bcl-2^Flox/Flox^ mice generally had 5 to 6 retinal arteries. In contrast, both Bcl-2^PC^ and Bcl-2^AC^ mice had significantly fewer retinal arteries and subsequently fewer veins (Fig. 2). We also noted that Bcl-2^PC^ mice have similar second and third order branching compared to Bcl-2^Flox/Flox^ mice. Bcl-2^AC^ mice demonstrated a modest decrease in second order branching compared to Bcl-2^Flox/Flox^ littermates (Fig. 2). Thus, lack of Bcl-2 in pericytes or astrocytes decreased retinal artery and vein numbers.

### Decreased vascularization of the central retina in Bcl-2^PC^ mice

**Figure 3:**
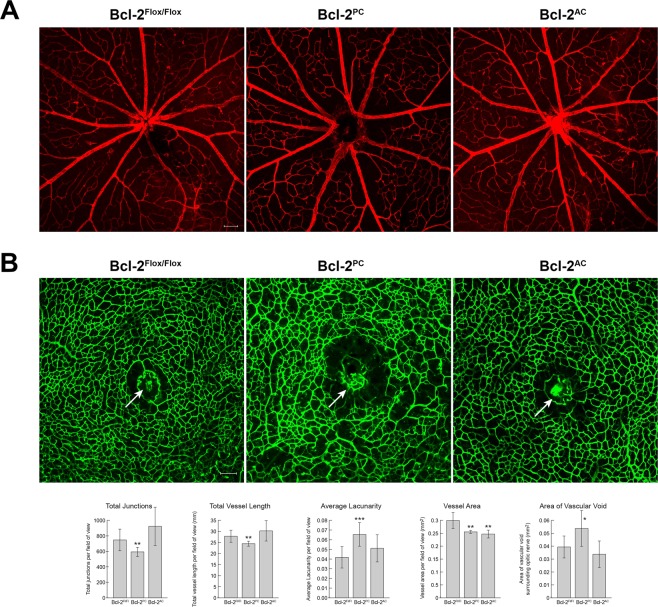
Aberrant vascularization of the central retina in Bcl-2^PC^ mice. In Panel A, retinas from P10 mice were wholemount stained with anti-collagen IV. Photomicrographs were taken of retinal arteries near the optic nerve. In Panel B, retinas from P10 mice were wholemount stained with Isolectin B4-FITC and the deep vascular plexus imaged surrounding the optic nerve. The arrow points to the optic nerve. Scale bar = 100 µm (n = 5). Please note symmetrical branching of retinal arteries from Bcl-2^Flox/Flox^ and Bcl-2^AC^ mice but disorganized appearance of the vasculature in the retinas from Bcl-2^PC^ mice and void near optic nerve.

Our prior studies conducted on mice lacking expression of Bcl-2 in endothelial cells showed aberrant patterning of the central retinal vasculature^12^. Since we observed primary branching off retinal arteries close to the optic nerve in Bcl-2^PC^ and Bcl-2^AC^ mice we next assessed whether vascularization of the central retina was affected in these mice (Fig. 3A). Although vascularization of the central retina was similar in Bcl-2^Flox/Flox^ and Bcl-2^AC^ mice, Bcl-2^PC^ mice demonstrated bifurcation of the retinal artery farther from the optic nerve thus leaving a central retinal vascular void (Fig. 3A). Thus, Bcl-2 expression in pericytes may aid appropriate positioning of vessels entering the central retina and appropriate retinal vascularization.

Formation of the superficial retinal vascular layer is followed by sprouting of these vessels deep into the retina forming the deep vascular plexus. To assess whether the retinal deep vascular plexus formed in mice lacking expression of Bcl-2 in perictyes or astrocytes we stained retinas from P10 Bcl-2^Flox/Flox^, Bcl-2^PC^ and Bcl-2^AC^ mice with Isolectin B4. The deep vascular plexus did form in all these mice, albeit to different extents (Fig. 3B). The capillary network in deep vascular plexus surrounding the optic nerve had a similar fairly uniform appearance in Bcl-2^Flox/Flox^ and Bcl-2^AC^ mice. This is consistent with the similar average lacunarity, vessel length and junctions (Fig. 3B). In contrast, the deep vascular plexus in retinas from Bcl-2^PC^ mice had a more disorganized or disjointed appearance, although well-developed areas similar to that observed in Bcl-2^Flox/Flox^ littermates were interspersed. The central vascular void described in Fig. 3A is also apparent around the optic nerve in the deep vascular plexus in retinas from Bcl-2^PC^ mice and is significantly increased compared to Bcl-2^Flox/Flox^ mice. We also noted the vessel area was decreased in both Bcl-2^PC^ and Bcl-2^AC^ mice compared to their Bcl-2^Flox/Flox^ littermates (Fig. 3B). Therefore, Bcl-2 expression in pericytes may aid the proper formation of the deep vascular plexus.

### Development of the superficial vascular layer of the retina is delayed in Bcl-2^PC^ mice

**Figure 4:**
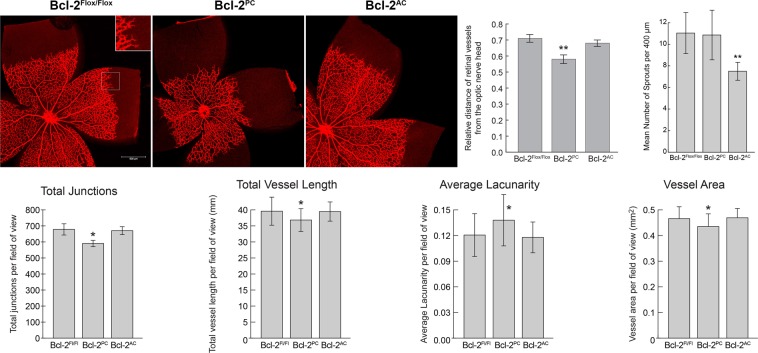
Bcl-2^PC^ mice demonstrate reduced distance of migrating front from the optic nerve. Retinas from P5 mice were wholemount stained with anti-collagen IV and the distance retinal vessels spread relative to the radius of the retina assessed in each quadrant (n = 5; **P < 0.01). The inset magnifies (200%) the sprouting at the vascular front in the boxed area. Tip cell and junction numbers as well as lacunarity, vessel length and vessel area were also determined. Scale bar = 500 µm. Please note that the distance the retinal vessels spread was decreased in Bcl-2^PC^ mice compared to Bcl-2^Flox/Flox^ and Bcl-2^AC^ mice.

Since we had observed significant changes in endothelial cell, pericyte and artery numbers in Bcl-2^PC^ and Bcl-2^AC^ mice, we next examined the expansion pattern of the retinal superficial vascular layer. Our prior studies showed that although global lack of expression of Bcl-2 delayed progression of the retinal superficial vascular layer, lack of expression of Bcl-2 only in endothelial cells did not^11,12^. Here we stained retinas from P5 pups Isolectin B4 to visualize the retinal superficial vascular layer. As shown in Fig. 4, the retinal superficial vascular layer had spread a similar distance from the optic nerve head in retinas from P5 Bcl-2^Flox/Flox^ and Bcl-2^AC^ pups. In contrast, in retinas from P5 Bcl-2^PC^ pups the distance from the migrating front to the optic nerve was reduced compared to Bcl-2^Flox/Flox^ littermates (Fig. 4). We also examined vascular complexity and demonstrate increased lacunarity in retinas from Bcl-2^PC^ mice compared to Bcl-2^Flox/Flox^ littermates as well as decreased vessel area, vessel length and total number of junctions (Fig. 4). Bcl-2^AC^ mice demonstrated a significant decrease in tip cell sprouts compared to Bcl-2^Flox/Flox^ littermates (Fig. 4). Thus, the data presented here indicate that Bcl-2 expression in pericytes plays an important role during progression of the retinal superficial vascular layer^12^.

### Increased apoptosis and proliferation in retinas from Bcl-2^PC^ mice

**Figure 5:**
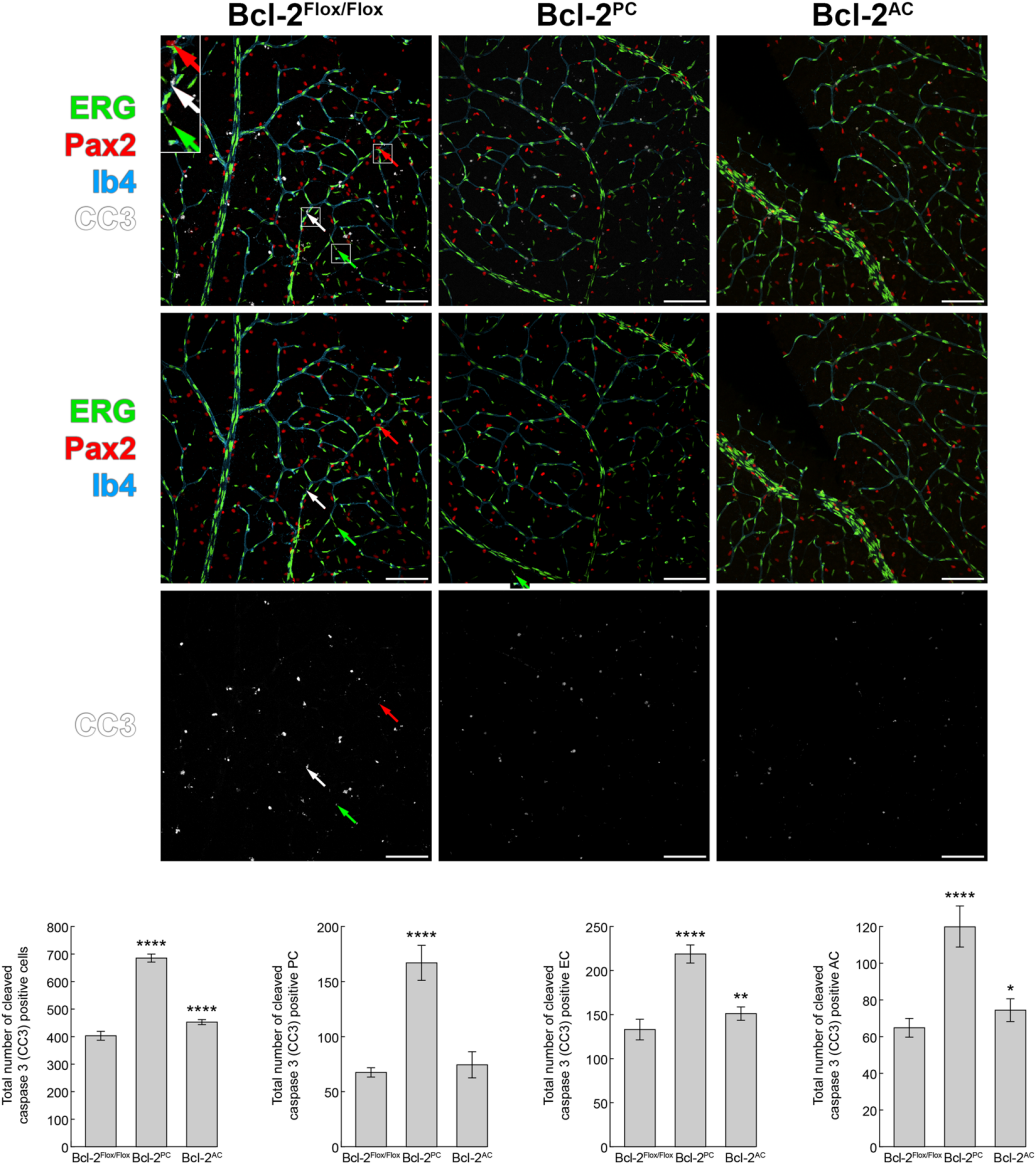
Increased apoptosis in retinas from Bcl-2^PC^ and Bcl-2^AC^ mice. Retinas from P14 Bcl-2^Flox/Flox^, Bcl-2^PC^ and Bcl-2^AC^ mice were wholemount stained with anti-cleaved caspase 3 (white), anti-ERG (green), anti-Pax2 (red) and Isolectin-B4-FITC (blue). The cleaved caspase-3 positive cells associated with the vasculature were quantified throughout the entire retina that were anti-ERG positive (endothelial cells), anti-Pax-2 positive (astrocytes) or non-labeled (pericytes). Arrows point to apoptotic endothelial cell (green), astrocyte (red) and pericyte (white). The inset is a 200% magnification of the boxed areas of interest. Scale bar = 100 µm. The mean number of cleaved caspase 3 positive endothelial cells, pericytes and astrocytes associated with the vasculature of each retina is denoted in the bar graph. Please note macrophages distinguished by their intense staining and large size, were not counted in the total number of capase 3 positive cells (n = 6, ***P < 0.001, ****P < 0.0001).

**Figure 6:**
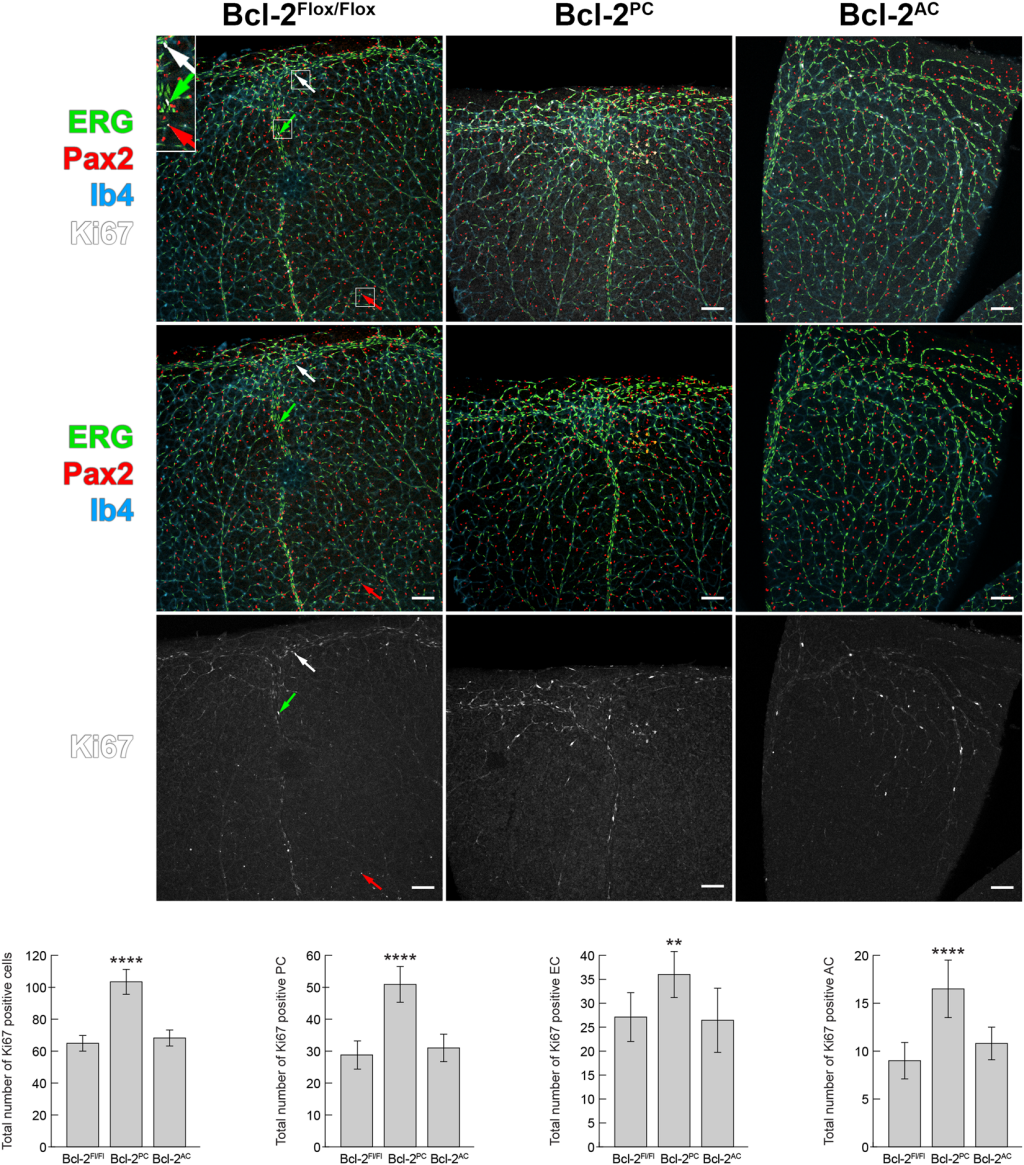
Increased proliferation in retinas from Bcl-2^PC^ mice. Ki-67 staining was used to visualize proliferating cells in P14 Bcl-2^Flox/Flox^, Bcl-2^PC^ and Bcl-2^AC^ mouse retinas. Retinas were co-stained with anti-ERG (green: endothelial cells), anti-Pax2 (red: astrocytes), unstained (pericytes) and Isolectin-B4 (blue: vasculature). Throughout the entire retina the mean number of Ki-67 positive endothelial cells, perictyes and astrocytes were then determined for each retina. Arrows point to proliferating cells that were anti-ERG positive (endothelial cell: green arrow), anti-Pax2 (astrocyte:red arrow) or pericytes (non-staining: white arrow). Insets are a magnification (200%) of the boxed areas of interest. Scale bar = 100 µm. Please note that the number of proliferating vascular cells were increased in retinas from Bcl-2^PC^ mice compared with their Bcl-2^Flox/Flox^ counterparts (n = 6, ^****^P < 0.0001).

Not only does Bcl-2 regulate apoptosis, its expression also modulates proliferation^16^. Here we assessed the level of apoptosis by anti-cleaved caspase 3 staining and proliferation by anti-Ki-67 staining of retinas from P14 mice. The vasculature was en face stained with Isolectin B4. Endothelial cells and astrocytes were immunolabeled with anti-ERG and anti-Pax2 antibodies, respectively. While pericytes were not directly labeled, they were recognized when cleaved caspase 3 or Ki-67 positive cells overlapped with the vasculature (Isolectin B4 positive vessels) but not with either ERG or Pax2 positive cells. Bcl-2^PC^ mice demonstrated increased endothelial cell, pericyte and astrocyte apoptosis compared to Bcl-2^Flox/Flox^ mice (Fig. 5). Bcl-2^AC^ mice only had increased astrocyte and endothelial cell apoptosis (Fig. 5). Bcl-2^PC^ mice also demonstrated increased retinal endothelial cell, pericyte and astrocyte proliferation compared to Bcl-2^Flox/Flox^ mice, as shown by anti-Ki-67 staining (Fig. 6). In contrast, no significant change in proliferation was observed in retinas from Bcl-2^AC^ mice compared with control littermates (Fig. 6). Thus, pericyte apoptosis and proliferation was significantly impacted by the lack of Bcl-2 expression.

### Mice conditionally lacking Bcl-2 demonstrated similar levels of ischemia-driven retinal neovascularization

**Figure 7:**
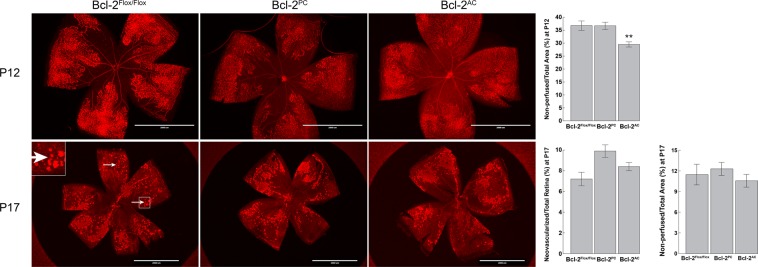
Hyperoxia-driven retinal neovascularization is not impacted by conditional lack of Bcl-2 expression. Mice were exposed to a cycle of hyperoxia and room air (OIR). To visualize the vasculature, retinas from P12 and P17 Bcl-2^Flox/Flox^ and conditional Bcl-2 littermates were wholemount stained with anti-collagen IV. At P12 and P17 the area of vessel obliteration relative to the whole retina was quantified (n = 6, **P < 0.01). In the lower panel at P17 the area of neovascularization relative to the area of the retina was quantified (n = 6, P > 0.05). Arrows point to neovascular tufts, in which an area of interest in noted and magnified 300% in the inset. Please note similar levels of neovascularization in Bcl-2^Flox/Flox^ mice their littermates conditionally lacking Bcl-2. Scale bar = 2000 µm.

Developmental retinal vascularization is coupled to oxygen requirements of the neuroretina. This makes the neuroretina inherently sensitive to changes in oxygen levels during the early stages of development, as evidenced by aberrant retinal vascularization in preterm infants with retinopathy of prematurity. Since we had previously observed a requirement for global Bcl-2 expression^11^, but not Bcl-2 expression specifically in endothelial cells^12^ for ischemia-driven retinal neovascularization, here we examined whether expression of Bcl-2 in pericytes or astrocytes was essential for neovascularization in a mouse OIR model. P7 Bcl-2^Flox/Flox^, Bcl-2^PC^ and Bcl-2^AC^ pups were exposed to 75% oxygen for 5 days (P12) and then sent back to room air for an additional 5 days (P17). Exposure to hyperoxia promoted loss of existing vessels in P12 Bcl-2^Flox/Flox^, Bcl-2^PC^ and Bcl-2^AC^ mice, although Bcl-2^AC^ mice demonstrated less retinal vessel loss (Fig. 7). Upon return to room air the retina remedies its ischemia by promoting (i) physiologic intraretinal angiogenesis, which vascularizes the hyperoxia-induced vaso-obliterated retina, and (ii) pathologic intravitreal angiogenesis, which forms vascular tufts (neovascularization) on the surface of the retina towards the vitreous. The formation of these neovascular tufts reach a maximum at P17 before they start to regress. At P17, the retinal capillary-free area was similar between Bcl-2^Flox/Flox^ mice and those conditionally lacking Bcl-2 in pericytes or astrocytes (Fig. 7). This suggests that ischemia-induced physiologic intraretinal angiogenesis was not affected by lack of Bcl-2 expression in either pericytes or astrocytes. Additionally, similar levels of neovascularization were observed in P17 Bcl-2^Flox/Flox^, Bcl-2^PC^ and Bcl-2^AC^ mice (Fig. 7). Thus, lack of expression of Bcl-2 in pericytes or astrocytes did not impact neovascularization associated with OIR.

### Lack of expression of Bcl-2 in pericytes reduces choroidal neovascularization

**Figure 8:**
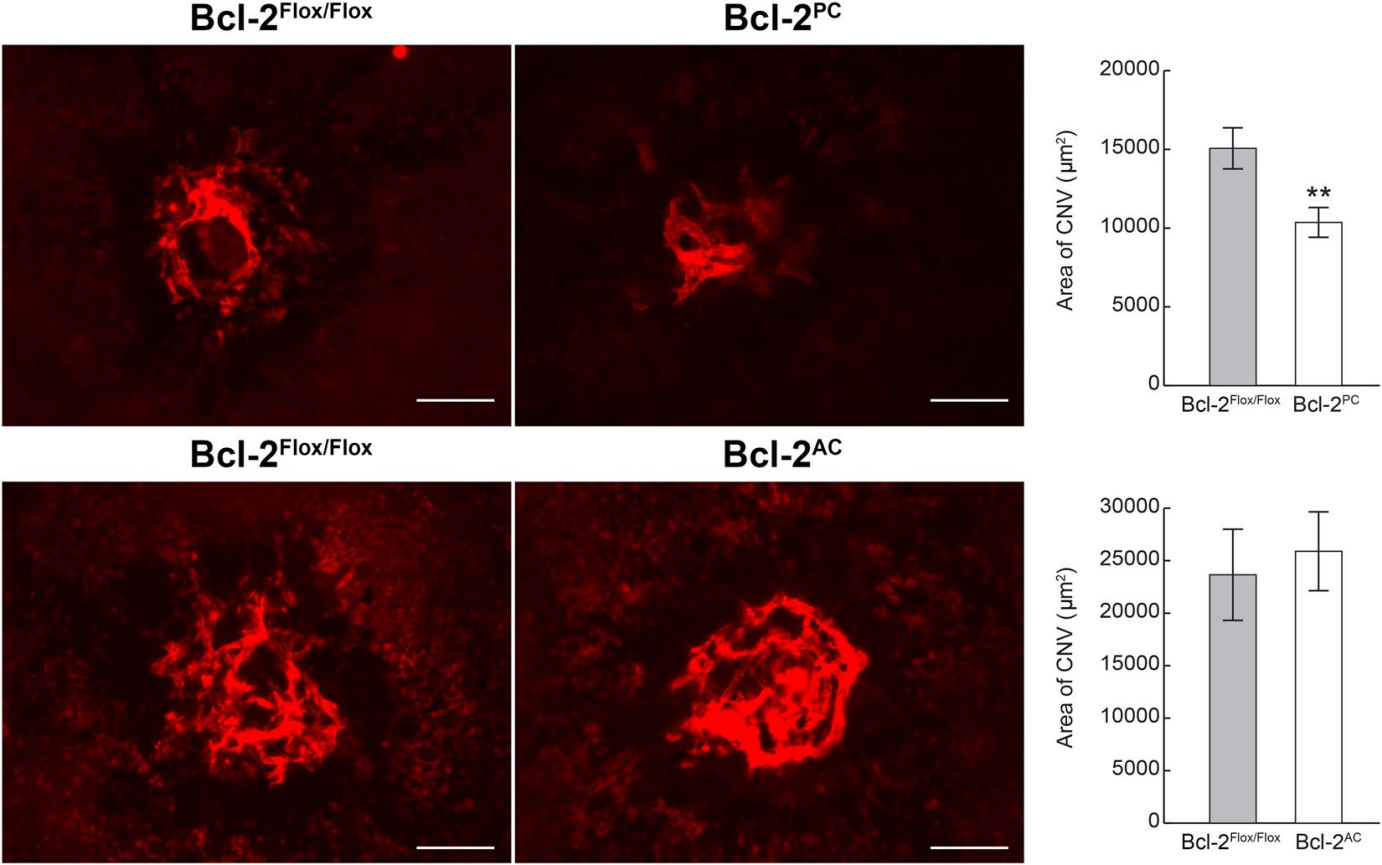
Lack of Bcl-2 expression in pericytes attenuates choroidal neovascularization. Six-week old mice underwent laser photocoagulation-induced rupture of Bruch’s membrane and 14 days later the eyes were sectioned at the equator. The anterior half/vitreous/retina was removed and the remaining eye tissue stained with anti-ICAM-2. The intensity of staining of the area of neovascularization was quantified using Image J (^**^P < 0.01; n = 10). Scale bar = 100 µm.

The choriocapillaris nourishes the outer retina. Dysfunction of this circulation plays roles in the development of age-related macular degeneration and CNV. Here we assessed whether Bcl-2 expression in pericytes or astrocytes could impact choroidal neovascularization in a laser induced model of CNV. Using laser photocoagulation, Bruch’s membrane was ruptured in 6-week old mice. Next, the area of CNV was determined 2 weeks later by ICAM-2 staining. Bcl-2^PC^ mice showed significantly less CNV than Bcl-2^Flox/Flox^ mice (Fig. 8). Bcl-2^Flox/Flox^ and Bcl-2^AC^ mice demonstrated similar levels of CNV. Thus, Bcl-2 expression in pericytes facilitates CNV.

## Discussion

Bcl-2 was initially identified due to its translocation in follicular lymphoma and was subsequently found to inhibit apoptosis rather than increase proliferation^17^. It is widely expressed during development, but its expression becomes restricted upon maturation in many tissues^18^. Bcl-2 expression also influences VEGF expression, extracellular matrix production, cell adhesion and migration which may be why its lack of expression has far reaching consequences^13,14,19–29^, including aberrant organ development^30^. We have reported that both Bcl-2 and Bim proteins play important roles in vascular development, remodeling and pruning in various organs including retina, lung and kidney^11,13,31^. Over-expression of Bcl-2 in endothelial cells not only improves formation of blood vessels but also promotes progressive maturation of vasculature by recruitment of perivascular supporting cells including vascular smooth muscle cells and pericytes^32,33^. However, little is known as to what impact Bcl-2 expression in pericytes or astrocytes has during retinal vascular development or pathological neovascularization.

Independent of its role in preventing apoptosis, Bcl-2 can act as an angiogenic factor by promoting expression of VEGF and HIF-1α^19,20^. In the absence of Bcl-2, capillary morphogenesis and aortic ring sprouting is attenuated^13,25^. This may be due to less endothelial nitric oxide synthase (eNOS), an important downstream effector of proangiogenic signaling^25^ or perhaps increased oxidative stress in the absence of Bcl-2 the relief of which restores capillary morphogenesis and sprouting defects^13,25^. Here we show that lack of expression of Bcl-2 in pericytes, but not astrocytes, attenuates CNV. Given astrocytes are not reported to be associated with the choroidal vasculature the inability of mice lacking Bcl-2 expression in astrocytes to attenuate CNV is not unexpected. We have also noted CNV attenuation in Bcl-2+/− mice (Fig. S1) and mice lacking Bcl-2 expression in endothelial cells (Bcl-2^EC^ mice)^12^. Thus, decreasing Bcl-2 expression may be a useful approach to attenuate CNV.

Retinopathy of prematurity is a vision-threatening eye disease that results from exposure of preterm infants to high oxygen. The OIR mouse model mimics this disease, allowing us to study in detail how to better manage hypersensitivity of the developing retinal vasculature to hyperoxia-driven vascular apoptotic regression and subsequent ischemia-driven retinal neovascularization^34^. Our prior studies in mice globally lacking Bcl-2 showed reduced retinal neovascularization even though the level of vaso-obliteration at P12 was similar to control littermates after exposure to OIR mouse model^11^. Here we show that lack of expression of Bcl-2 in pericytes or astrocytes did not impact neovascularization or intraretinal vascular regeneration with OIR.

Retinal vascularization follows a scaffolding laid out by astrocytes^35^ with pericytes covering and stabilizing nascent vessels. Global loss of Bcl-2 not only decreased retinal artery number but also retinal vascularization, consistent with delayed spreading of the superficial layer, and decreased endothelial cell and pericyte numbers^11^. However, Bcl-2^EC^ mice only recapitulated a portion of these retinal deficits^12^, suggesting that Bcl-2 expression in other retinal vascular cells make a significant contribution during development. Here we show that numbers of pericytes and endothelial cells in the retina were significantly reduced in Bcl-2^PC^ and Bcl-2^AC^ mice, even to an extent greater than we previously observed in Bcl-2^EC^ mice^12^. However, astrocytes numbers were similar for Bcl-2^Flox/Flox^, Bcl-2^PC^ and Bcl-2^AC^ mice. Furthermore, pericyte numbers were lower in Bcl-2^AC^ than Bcl-2^PC^ mice demonstrating the reliance each vascular cell type has on each other for proper retinal vascularization.

Astrocytes play a critical role during retinal vascularization, migrating out ahead of the vascular network. This sets a scaffolding for the forming new vessels to follow which may be why decreased numbers of pericytes and endothelial cells were noted in Bcl-2^AC^ mice even though astrocyte numbers were similar. It is possible that decreased numbers of retinal pericytes and endothelial cells may reflect an increase in apoptosis of these cells without subsequent increased proliferation, as we normally observe in the absence of Bcl-2^11,12^. Given the role Bcl-2 has in influencing migration and adhesion its lack of expression in retinal astrocytes may disrupt the intercommunication between vascular cell types leading to decreased endothelial cell and pericyte numbers.

Our previous studies demonstrated aberrant patterning of the central retinal vasculature in Bcl-2^EC^ mice^12^. Here we examined the impact of Bcl-2 expression in pericytes or astrocytes had on the central retinal vasculature. Lack of vascularization in the central retina was only observed in Bcl-2^PC^ mice, where vessels appeared to enter and bifurcate near the disc periphery. Such aberrant vascularization in the central retina is similar to that observed in papillorenal syndrome in which retinas lack central vessels that should have emerged from the periphery of the nerve papilla as well as renal hypoplasia^36–38^, as we observed in global Bcl-2 −/− mice. However, unlike the global Bcl-2 −/− mice we did not observe renal hypoplasia in Bcl-2^PC^ mice as evidenced by similar kidney to body weight ratios of 3 week old Bcl-2^Flox/Flox^ and Bcl-2^PC^ mice (Fig. S2). The data presented here and our previous studies indicate an important role for Bcl-2 expression in endothelial cells and pericytes for proper patterning of the central vessels in the retina. Thus, proper numbers of pericytes and their function is important not only during development but also to maintain proper vascular function^8–10^.

## Materials and Methods

### Ethics statement

The experiments shown here were performed in accordance to the Association for Research in Vision and Ophthalmology Statement for the Use of Animals in Ophthalmic and Vision Research. The protocols were approved by the Institutional Animal Care and Use Committee of the University of Wisconsin School of Medicine and Public Health approved. Euthanasia by CO2 asphyxiation was done according to approved protocols.

### Animals

Gfap-cre (B6.Cg-Tg (Gfap-cre) 73.12Mvs/J; Jackson Laboratory stock number 012886), Tg(Pdgfrb-cre)^*45Vli*^ and Bcl-2^Flox/Flox^ (bcl2tmlIrt/J; Jackson Laboratory stock number 008882) were kept at the University of Wisconsin animal facilities. Genotyping of the Tg(Pdgfrb-cre)^*45Vli*^ mouse line was done using the following primers: 5′-GCATTTCTGGGGA TTGCTTA-3′ and 5′-CCCGGCAA AACAGGTAGTTA-3′^39^, GFAP-cre mice with 5′-TCCATA AAGGCCCTGACATC-3′ and 5′-TGCGAACCTCATCACTCGT-3′ and Bcl-2^Flox/Flox^ mice with 5′-GCCCACCAT CTAAAGAGCAA-3′ and 5′- GCATTT TCCCACCACTGTCT-3′. We bred mice homozygous for the floxed allele (Bcl-2^Flox/Flox^) with Tg(Pdgfrb-cre)^*45Vli*^ or Gfap-cre mice to generate heterozygous mice for the Bcl-2^Flox/Flox^ allele that expressed Pdgfrb-cre or Gfap-cre. These mice were bred and the offspring genotyped as explained above to generate mice homozygous for the Bcl-2^Flox/Flox^ allele that also expressed the desired cre. We sustained the colony by breeding mice homozygous for the Bcl-2^Flox/Flox^ allele that were cre-expressing to mice homozygous for the Bcl-2^Flox/Flox^ allele and genotyping. Mice homozygous for the Bcl-2^Flox/Flox^ allele that express Pdgfrb-cre are referred to as Bcl-2^PC^ mice and those expressing Gfap-cre are referred to as Bcl-2^AC^ mice. Bcl-2^Flox/Flox^ mice are referred to as control littermates at times. To validate specificity of Cre-mediated excision, we generated mice carrying a conditional Tomato allele and Pdgfrb-cre or Gfap-cre. We noted Tomato expression in retinal pericytes/vascular smooth muscle cells^40^, as we previously described for astrocytes (Fig. S3). In all experiments, male and female mice were used.

For OIR mouse model, 7-day-old (P7) pups with dams were exposed to an atmosphere of 75 ± 0.5% oxygen for 5 days with the incubator temperature kept at 23 ± 2 °C, and oxygen was continuously monitored with a PROOX model 110 oxygen controller (Reming Bioinstruments Co., Redfield, NY). Retinal wholemounts were prepared after mice were sent back to room air for 5 days^11,31^.

### Trypsin-digested retinal vessel preparation

Enucleated eyeballs from P21 or P42 mice were fixed in 4% paraformaldehyde for at least 24 h. Then the eyeballs were bisected equatorially removing the whole retina under a dissecting microscope. Retinal trypsin digests were prepared and stained as previously described^11,40^. We used nuclear morphology to tell apart pericytes from endothelial cells. The number of pericytes and endothelial cells on retinal capillaries was determined masked by counting the number of nuclei per field of view under the microscope at a magnification of x400. Counts were performed on vasculature that corresponds to the middle (mid-zone) of the retina by counting the number of pericytes and endothelial cells in four fields of view from the four quadrants of each retina.

### Visualization of retinal vasculature

Eyeballs were enucleated and briefly fixed in 4% paraformaldehyde (10 min on ice) then placed for at least 24 h in 70% methanol at −20 °C as we previously described^11,12,40^. Blocked retinas were kept in blocking solution with anti-collagen IV (Millipore, Burlington, MA, AB756P)(diluted 1:250 in blocking solution (PBS containing 20% normal goat serum, 20% fetal calf serum)) or anti-hPax-2 (R&D systems, AF3364; diluted 1:100) at 4 °C overnight. The retinas were then incubated with an appropriate secondary antibody as required, mounted on a slide, viewed by fluorescence microscopy and images captured in digital format using a Zeiss microscope (Carl Zeiss, Chester, VA). For quantitative analysis of vascular networks (average lacunarity, total junctions, total vessel length, vessel area) the AngioTool software was used^41^. Image J was used to assess the area of vascular void near the optic nerve. For OIR studies, the central capillary dropout area was quantified (percentage of the whole retina area) from the digital images in masked fashion using Axiovision software (Carl Zeiss, Chester, VA). The method of Stahl etal was used for quantification of vitreous neovascularization^42^.

### Laser induced choroidal neovascularization (CNV)

CNV was caused after rupturing the Bruch’s membrane of 6 week old mice by laser photocoagulation. The day of laser photocoagulation was considered day 0. A mix of ketamine hydrochloride (80 mg/kg) and xylazine (10 mg/kg) was used to anesthetize mice and then a drop of tropicamide (1%) was used to dilate their pupils. A handheld cover slip was used as a contact lens to facilitate viewing the retina and a slit lamp delivery system of an OcuLight GL diode laser (Iridex, Mountain View, CA) was used to locate the 9, 12, and 3 o’clock positions of the posterior pole of each eye for laser photocoagulation (75 µm spot size, 0.1 sec duration, 120 mW). Fourteen days later the eyes were enucleated, fixed in 4% paraformaldehyde, and then washed in PBS which was followed by sectioning the eye at the equator, and the retina, the virous and the anterior half removed. The rest of the eye was then submerged in blocking buffer (20% normal goat serum and 5% fetal calf serum in 1xPBS) for 30–60 minutes. Then anti-ICAM-2 (BD Pharmagen, #553326) was prepared (1:500) in 1xPBS with 20% normal goat serum and 20% fetal calf serum at 4 °C overnight. This was followed by washing the tissues in 1xPBS before adding the the appropriate secondary antibody and keeping the samples staining for 2–4 hours on the shaker at room temperature. The retinal pigment epithelium-choroid-sclera complex was flatmounted on a slide, viewed by fluorescence microscopy and images were captured in digital format using a Zeiss microscope (Zeiss, Chester, VA). The total area (in µm^2^) of CNV associated with each laser burn was measured using Image J software (National Institute of Mental Health, Bethesda, MD; http://rsb.info.nih.gov/ij/).

### Smooth muscle actin (SMA) staining

The eyeballs were enucleated and washed from blood in 1xPBS and then fixed in 4% paraformaldehyde for 5 minutes before washing them again in 1xPBS and archiving them methanol under −20 °C until use. The day of staining, the eyeballs were rehydrated in 1xPBS for 30–60 minutes before the retinas were dissected in 1xPBS. Intact retinas were then blocked (20% normal goat serum, 50% fetal calf serum (FCS) and 0.1% Triton X-100 in 1xPBS) and incubated with anti-SMA-FITC (1:500; Sigma, #F3777 diluted in 20% goat serum, 20% fetal calf serum and 0.1% Triton X-100). The retinas were then washed in 1xPBS and flatmounted and imaged. The number of arteries and veins per retina were quantified.

### Formation of the Retinal superficial vascular layer

Eyes from P5 mice were collected and fixed in 4% paraformaldehyde (3–10 minutes) and stored at −20 °C in methanol until use. Next, the dissected retinas were washed in 1xPBS for 30 minutes then followed by a 30 minutes in 3% paraformaldehyde and then washed three times in 1xPBS. To assess the spreading of the superficial layer retinas were wholemount stained with anti-collagen IV and mounted with Fluormount-G (Southern Biotech, Birmingham, AL, #0100-01). For quantification, the distance the retinal vessels had spread from the optic nerve head to the periphery of the retina relative to the entire retinal radius was determined for each quadrant of the retina at P5 which was then recorded as an average for each retina. Tip cell sprouts were determined as we previously described^31^. The distance the superficial layer had spread was quantified from digital images taken on an EVOS microscope as we previously described^12^.

### Apoptosis and proliferation

Eyes from P14 mice were fixed in 4% paraformaldehyde then stored in methanol (−20°) until use, as we previously described [10]. Dissected retinas were placed in PBS for 30 minutes. The retinas were then incubated in 0.5% Triton X-100 in 1 x PBS for 60 minutes and then washed 3 times in 1 x PBS for 5 minutes. The retinas were then kept in blocking solution (1% BSA, 0.3% Triton X-100 in 1 x PBS) before the addition of primary antibodies. Goat anti-human Pax2 (1:200; R&D Systems #AF3364) and rabbit anti-ERG (EPR3864) (1:200; abcam, Cambridge, UK, #ab92513) antibodies were prepared in blocking solution and then added to the retinas. All rocking steps were done at 4 °C. Retinas were incubated with the primary antibodies for 72 hours while rocking. Retinas were then washed three times in 1 x PBS for 5 minutes each. Next, the retinas were incubated with the appropriate secondary antibody in blocking buffer (1:500; Jackson ImmunoResearch Laboratories) overnight rocking. The retinas where then washed twice in blocking solution with normal rabbit IgG (1:10; Millipore #Ni01-100µg) and kept rocking for 24 hours. Anti-cleaved caspase-3 Alexa Fluor 647(1:50; Cell Signaling clone D3E9 # 9602) or anti-Ki-67 Alex Fluor 647 (1:50; Cell Signaling clone D3B5 #12075) were diluted in blocking solution with normal rabbit IgG (1:10) and rocked for 48 hours. The retinas were co-stained with biotinylated-Isolectin B4 (1:100; Vector Labs #B-1205)) for 6 hours. After washing in blocking solution three times (20 minutes each) at room temperature on a rocker, streptavidin-Dylight 405 conjugate (1:500; Jackson ImmunoResearch Laboratories #016-470-084) in blocking solution was added and incubation continued for 2 hours. The retinas were next washed in 1 x PBS three times on a rocker for 24 hours total. Mounting was accomplished with mounting medium (Southern Biotech, #0100-01). The numbers of cleaved caspase-3 or Ki-67 positive endothelial cell (ERG positive), pericyte (cells associated with the blood vessels that did not stain positive for ERG or Pax2) and astrocytes (Pax2 positive) on the blood vessels were determined per retina.

### Statistical analysis

We evaluated statistical differences between groups with an ANOVA with Tukey’s Multiple Comparison Test. Mean ± standard deviation is shown. We then confirmed the comparison between Bcl-2^Flox/Flox^ and Bcl-2^PC^ or Bcl-2^Flox/Flox^ and Bcl-2^AC^ with a t-test. Mean ± standard deviation is shown. P < 0.05 was considered significant.

## Supplementary information

Figure S1-3
