# Supplementary material for: Bcl-2 Expression in Pericytes and Astrocytes Impacts Vascular Development and Homeostasis
Source: Sci Rep. 2019 Jul 4;9:9700. doi: 10.1038/s41598-019-45915-4 (PMC6609701; doi:10.1038/s41598-019-45915-4)
Supplement: Supplementary file 1 — Figure S1-3 [file 41598_2019_45915_MOESM1_ESM.docx]

**Supplementary Information**

**Bcl-2 Expression in Pericytes and Astrocytes Impacts Vascular Development and Homeostasis**

Ismail Zaitoun^1^, Catherine M. Wintheiser^1^, Nasim Jamali^1,2^, Shoujian Wang^1^, Andrew Suscha^1^, Soesiawati R Darjatmoko^1^, Katherine Schleck^3^, Barbara A. Hanna^1^, Volkhard Lindner^4^, Nader Sheibani^1,2^ and Christine M. Sorenson^2,3^*

Departments of ^1^Ophthalmology and Visual Sciences, University of Wisconsin School of Medicine and Public Health, Madison, WI, USA, ^2^McPherson Eye Research Institute, University of Wisconsin School of Medicine and Public Health, Madison, WI, USA, ^3^Pediatrics, University of Wisconsin School of Medicine and Public Health, Madison, WI, USA, ^4^Center for Molecular Medicine, Maine Medical Center Research Institute, Scarborough, ME, USA

**^*^Address correspondence to:**

Christine M. Sorenson

University of Wisconsin School of Medicine and Public Health

Department of Pediatrics

McPherson Eye Research Institute

1111 Highland Avenue Rm 9451

Madison, WI 53705, USA

Tel.: 608-263-5831/Fax: 608-265-3397

E-mail: [cmsorenson@pediatrics.wisc.edu](mailto:cmsorenson@pediatrics.wisc.edu)

Ismail Zaitoun

University of Wisconsin School of Medicine and Public Health

Department of Ophthalmology and Visual Sciences

McPherson Eye Research Institute

1111 Highland Avenue Rm 9418

Madison, WI 53705, USA

Tel.: 608-265-3049

E-mail: iszaitoun[@wisc.edu](mailto:cmsorenson@pediatrics.wisc.edu)

**Figure S1. Attenuation of CNV in Bcl-2 +/- mice.** Choriodal neovascularization was induced in six week old wild-type (WT) and Bcl-2 +/- female mice by laser photocoagulation-induced rupture of Bruch’s membrane. After 14 days, the eyes were stained with anti-ICAM-2 to visualize the area of neovascularization (left panel). The right panel is a quantitation of the area of neovascularization (^***^P<0.05).


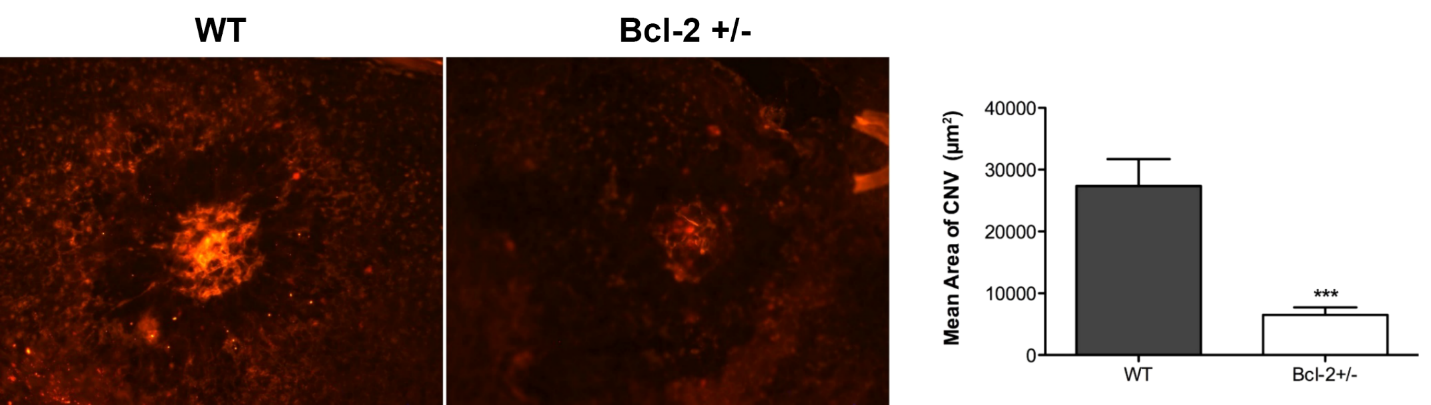


**Figure S2. Kidneys from Bcl-2^PC^ mice were not hypoplastic.** Mid-sagittal kidney sections from 3 week old Bcl-2^Flox/Flox^ and Bcl-2^PC^ mice were hematoxylin and eosin stained (left panel). Sections are representative of > 6 mice. Magnification was x200. The right panel demonstrates the kidney to body weight ratio at 3 weeks of age with means ± SD. Please note no significant difference in the kidney to body weight ratio was noted. Although kidneys from global Bcl-2 -/- mice were grossly cystic by 3 weeks of age, but here no renal cysts were noted in kidneys from 3 week old Bcl-2^PC^ mice.

**

**

**Figure S3:** **Astrocytes in Tomato^AC^ mice.** P21 Tomato^AC^ mice were perfused with FITC-wheat germ agglutinin to visualize the vasculature (green) with GFAP expressing cells (astrocytes; red). Scale bar equals 100 µm. Note astrocytes wrap around vessels.

**
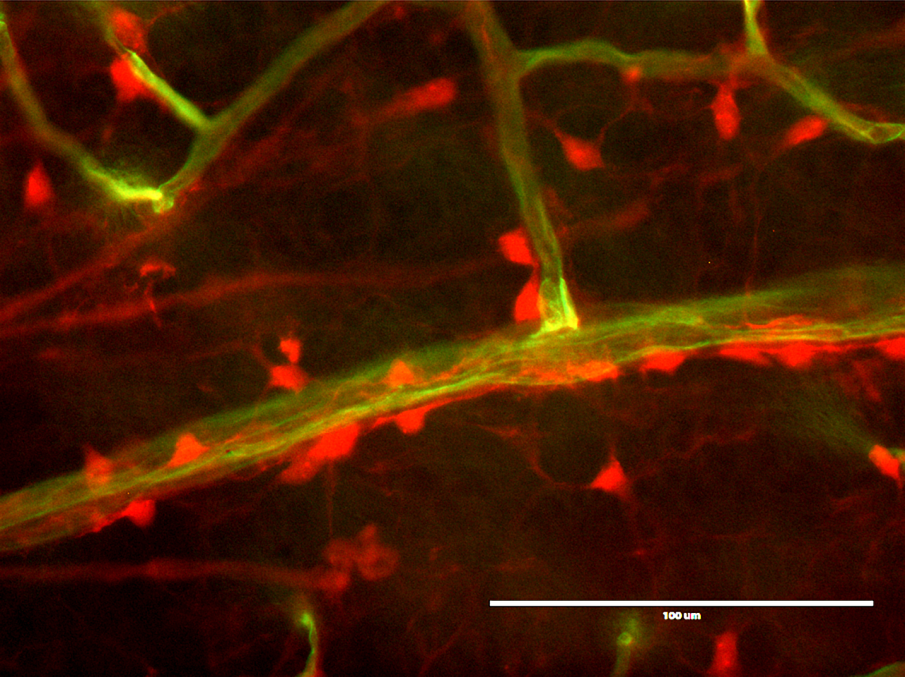
**
